# Long short-term attention memory (LSTAM): a global-feature-integrated model for joint moment prediction in human rehabilitation

**DOI:** 10.1038/s41598-026-42722-6

**Published:** 2026-03-17

**Authors:** Baoping Xiong, Yinghui Guo, Jie Lou, Zhenhua Gan, Jilin Zhang, Zhikang Su

**Affiliations:** 1 School of Computer Science and Mathematics, Fujian University of Technology, Fuzhou, 350116 China; 2 Fujian Zhikangyun Medical Technology Co., Ltd., Fuzhou, 350116 China

**Keywords:** Joint moment, Time series models, Biological signal, Global features, Local features, Human rehabilitation evaluation, Computational biology and bioinformatics, Engineering, Mathematics and computing, Neuroscience

## Abstract

Joint moments are critical parameters for evaluating human movement, and time-series models are widely used to predict them from biosignals. However, biosignals collected via accelerometers, gyroscopes, and electromyography (EMG) sensors are often susceptible to local features such as short-term fluctuations and noise, which hinders the models’ ability to effectively capture global features and weakens their capability to predict long-term trends. To address this issue, this paper proposes a long short-term attention memory (LSTAM) model that integrates global features. Our main contributions include the use of fast Fourier transform for spectral decomposition, multilayer perceptrons for nonlinear transformation, and convolutional modules to suppress the impact of local features in the sensor data. Additionally, an LSTM network enhanced with attention mechanisms is incorporated to dynamically focus on key temporal and frequency-domain patterns. We evaluated the proposed model on a publicly available dataset and compared its performance with existing methods, including LSTM, TCN, Conv2D, TimeMixer, xPatch, FFN, and TranSEMG. Experimental results show that the LSTAM model achieved a variance accounted for (VAF) of 0.907 ± 0.022 for hip flexion–extension (FE) and 0.927 ± 0.026 for hip abduction–adduction (AA); a root mean square error (RMSE) of 8.04 ± 2.27 (FE) and 5.56 ± 2.01 (AA); and a coefficient of determination (R^2^) of 0.908 ± 0.029 (FE) and 0.922 ± 0.030 (AA). These results demonstrate that LSTAM significantly outperforms existing models, offering a robust and efficient solution for joint moment prediction and human rehabilitation evaluation.

## Introduction

The joint moment is an important parameter for evaluating human motion and is widely used in clinical rehabilitation^1,2^, human–computer interaction^3,4^ and biomechanical research^5,6^. Since joint moment is difficult to measure directly in vivo, it is usually obtained through inverse dynamics methods^7^ and musculoskeletal models^8^. These methods rely on motion capture systems and ground reaction force measurement equipment to calculate the moment of specific joints, which requires precise anatomical models and a large amount of laboratory equipment, making the analysis process complicated and limited to the experimental environment^9^, hence greatly limiting the wide application of the joint moment research.

To solve this problem, in recent years, the prediction of joint moment using artificial neural networks (ANN)^10^ and deep learning models^11,12^ has become a research hotspot. These models can extract features from relevant biological signals of the human body and accurately predict joint moment in a non-invasive way, which greatly reduces resource consumption and improves the feasibility and extensiveness of the research^13^. Xiong et al.^14^ developed an ANN-based model that leverages both kinematic and kinetic data to predict lower extremity joint moments during various physical activities. Scherpereel et al.^15^ introduced a system that combines electromyogram (EMG) signals and instrumented insoles to estimate joint moments. The integration of multiple data sources improves prediction accuracy by leveraging both physiological and mechanical signals. Liu et al.^16^ developed a model that identifies muscle synergies from EMG signals and uses them to train a long short-term memory (LSTM) network to predict knee joint moments during walking. Jamaludin et al.^17^ used Transcranial motor-evoked potential (TcMEP) signals combined with machine learning to predict postoperative functional outcomes in patients undergoing lumbar spine surgery, demonstrating the feasibility of the approach in terms of sensitivity and specificity, and highlighting its potential for further improvement with larger datasets and diverse signal features. These methods effectively use EMG signals to predict joint moments, demonstrating the validity of EMG in this application. But none of them pays special attention to the impact of local features presented in such a complex biological signal on the model.

Surface electromyography (sEMG) signals not only reflect muscle activity but also provide reliable data for predicting joint moments^18,19^. However, during the acquisition of biosignals through hardware devices, data often contain varying degrees of noise or fluctuations, which can negatively impact the performance of predictive models^20,21^. To address the processing of biosignals, model research frequently includes a preprocessing phase to clean the raw data. Phinyomark et al.^22^ utilized wavelet transform to process sEMG signals, effectively reducing the influence of local features and improving model performance. Similarly, Englehart et al.^23^ employed principal component analysis (PCA) to denoise and extract features from sEMG signals, enhancing prediction accuracy. Bao et al.^24^ applied a third-order Butterworth bandpass filter (20–450 Hz) to remove low-frequency motion artifacts and high-frequency noise from the raw signal, thereby improving signal quality. They then used convolutional neural networks (CNN) to extract high-level features, followed by LSTM-KF for sequence regression, significantly improving the accuracy of hand movement estimation. These studies demonstrated that raw biosignals contain many unnecessary local features, and the methods employed to mitigate their influence often require manual parameter tuning (e.g., filter cutoff frequencies and Kalman filter noise covariance matrices)^25,26^ . In artificial intelligence models, feature extraction that includes unnecessary details, such as noise, can lead to inefficiencies in prediction. Therefore, leveraging models that automatically adjust parameters to effectively obscure the impact of local features while emphasizing global feature processing is becoming increasingly feasible in deep learning.

In addition, the complexity and nonlinear relationships in biological signal data often make traditional time series models inefficient in feature extraction and selection, and fail to fully utilize the useful information in the data^27^. TranSEMG developed by Chang et al.^28^ combines spatial and temporal sEMG features to enhance the local and global processing capabilities of biological signals, but still retains local features with noise, and there is still room for improvement in processing nonlinear signals. Similarly, BioMAT developed by Sharifi-Renani et al.^29^ uses a Transformer-based architecture to accurately predict joint movements in daily activities, but it lacks robustness to global features and has high model complexity. In time series analysis, global features refer to those that can capture long-term trends, periodic patterns, and general structural information throughout the time series. Unlike local features, global features focus more on the overall behavior of the time series and are usually extracted through frequency domain analysis, trend decomposition, or global statistics^28,30^. In addition, although the temporal convolutional network (TCN) model developed by Molinaro et al.^28^ can make good use of biological signals^31^, it may have limitations in processing long-term dependencies and non-stationary signals.

In order to more effectively utilize the nonlinear features and temporal dependencies in the global features of biological signals, this study proposes a LSTAM model for predicting joint moments. The first model converts the signal to the frequency domain, then uses a multi-layer perceptron (MLP) combined with Convolutional modules for nonlinear transformation to reduce the impact of local features, and combines the LSTM model to enhance the model’s processing of long-term dependencies of biological signals. Finally, the attention mechanism is used to enhance the model’s extraction of global features. In summary, the main contributions of this paper include:

1. The biological signal time series is converted to the frequency domain through fast Fourier transform, and the autoencoder (MLP combined with Convolutional modules) is used to reduce the influence of local features, leading to the performance of extracting global features directly optimized within the end-to-end model training framework.
2. The LSTM model is used to extract long-term dependency concerns, and the self-attention mechanism is combined to enhance the extraction of global features, thereby improving the prediction ability of the model.
3. The proposed model is evaluated on public databases to demonstrate the effectiveness and robustness of LSTAM in predicting joint moments from biological signals. Through comprehensive analysis and comparison with mainstream time series model methods, the results show that the proposed model achieves higher accuracy on various test sets with less training data. These evaluation results provide strong evidence for the effectiveness of our proposed model.

Previous studies on joint moments prediction using EMG signals, including the CNN-LSTM hybrid model^32^, the temporal convolutional network (TCN) method^33^, and the attention enhancement method^34^, mainly focus on local temporal correlations without fully addressing the interference of high-frequency noise and transient artifacts. The CNN-LSTM model used convolutional layers to extract short-term muscle activation patterns, but ignored the spectral features that encode global neuromuscular coordination. Similarly, the TCN model emphasized the use of dilated convolutions for multi-scale temporal modeling, but lacked an explicit mechanism to suppress high-frequency noise in the raw EMG signals, resulting in overfitting of transient fluctuations. Although the attention enhancement method introduced attention to reweighted temporal features, their framework is still limited to time domain representation, limiting its ability to distinguish persistent biomechanical patterns from local perturbations. In contrast, LSTAM systematically addresses these limitations through three innovations: fast Fourier transform (FFT)-based spectral decomposition explicitly captures global neuromuscular synergies in the frequency domain, which is less sensitive to transient noise^35^; the MLP-enhanced convolutional module performs nonlinear filtering to attenuate high-frequency artifacts while preserving biomechanically meaningful low-frequency components; and the attention mechanism dynamically prioritizes time-critical movement phases and frequency-specific coordination patterns, ensuring robust alignment between local muscle activations and global joint dynamics. The synergistic integration of time-frequency analysis and adaptive feature recalibration enables LSTAM to outperform traditional models in suppressing local feature interference.

The rest of this paper is organized as follows. In “Methodology” section introduces the proposed method. In “Experiment” and “Results” sections describe the details of the experiments and results. In “Discussion” section discusses the results and some limitations. In “Conclusion” section is the conclusion of this paper.

## Methodology

**Fig. 1:**
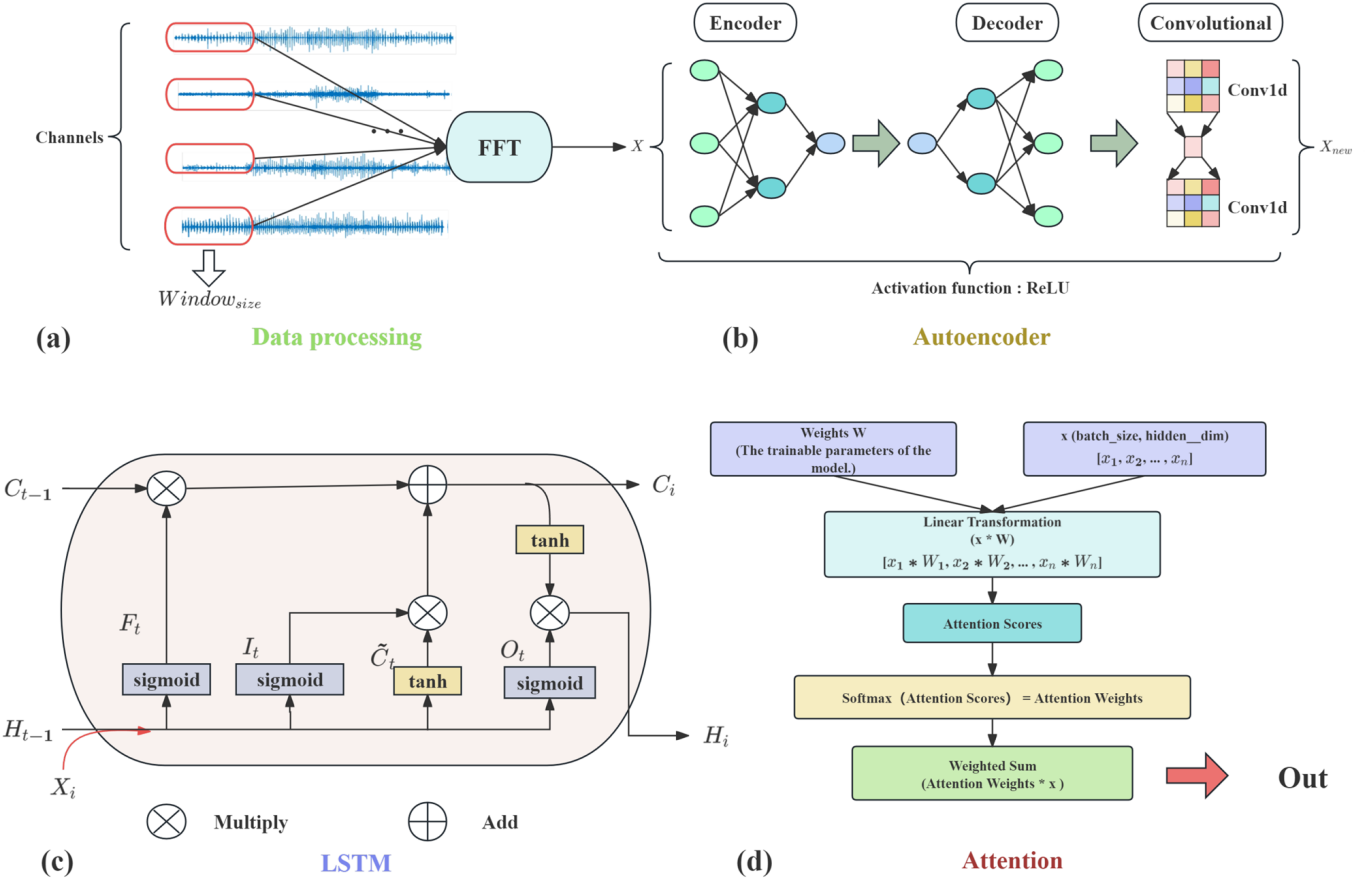
Schematic diagram of the LSTAM model.

The steps of predicting joint moments using the LSTAM model are illustrated in Fig. 1, and the corresponding parameters are listed in Table 1. To systematically extract global features from biological signals, we first apply a sliding window to segment the input signals, enabling the model to handle temporal variations. Each segment is then transformed to the frequency domain using FFT, which captures long-term spectral patterns that reflect global neuromuscular coordination. To reduce the interference of local fluctuations and noise, we apply an MLP combined with CNN, which selectively filters out high-frequency artifacts while preserving meaningful low-frequency components. Finally, an LSTM network enhanced with attention mechanisms integrates information across time steps, emphasizing critical movement phases and frequency-specific patterns. The synergy of these components—from frequency decomposition, nonlinear filtering, to temporal attention—ensures that the model effectively captures global features for accurate joint moment prediction. Detailed descriptions of each component are provided below.

**Table 1:** Parameters and variables in the LSTAM model, including inputs, outputs, LSTM states, gate matrices, and frequency- or autoencoder-derived features.

Parameter	Definition
*Channels*	Number of channels
$Windows_{size}$	Sliding window size
*FFT*	Fast Fourier transform
*X*	Input of MLP after fast Fourier transform
$X_{new}$	Parameters obtained after autoencoder
$C_{t-1}$	Memory unit of the previous time step
$H_{t-1}$	Hidden state of the previous time step
$F_t$	Forget gate
$I_t$	Input gate
$\tilde{C}_t$	Candidate memory unit
$O_t$	Output gate
$C_t$	Memory unit of current time step
$H_t$	Hidden state at the current time step
*x*	LSTM parameters obtained after extracting features
$W_i$	Weight matrix input to input gate
$W_f$	Weight matrix input to forget gate
$W_o$	Weight matrix input to output gate
$W_c$	Weight matrix input to candidate cell state
$U_i$	Weight matrix input to input gate
$U_f$	Weight matrix input to forget gate
$U_o$	Weight matrix input to output gate
$U_c$	Weight matrix input to candidate cell state
*t*	Input vector of current time step

### Data processing

The time series model requires sufficient data to ensure that the model can handle long-term dependency problems^36^. When dealing with prediction tasks using biological signals with less data, researchers often uses a sliding window to give the model more memory units to enhance the model’s feature extraction capabilities^37,38^. This study will use overlapping sliding windows to enable the LSTAM model to extract more features to deal with the problem of fewer biological signals under different modalities.

In addition, biological signals are nonlinear signals in the time domain, and it is difficult to detect periodic features and process noise^39^. FFT converts time domain signals into frequency domain. Frequency domain signals describe the intensity and phase of different frequency components in the time domain signal, and contain all the information of the time domain signal. Each frequency component corresponds to a periodic feature in the time domain signal^40^. Therefore, this study considers converting biological signals such as sEMG signals and joint angles into the frequency domain so that the model can better process global features and extract the features of related human body signals when actions occur.

### Autoencoder

During the process of acquiring biological signals, various uncontrollable factors often introduce interference such as noise and other local features^41^. These local features not only contribute minimally to subsequent analysis and applications but can also introduce misleading information, thereby diminishing data interpretability and prediction accuracy. To address this issue, this study introduces an Autoencoder architecture that combines MLP and CNN methodologies. The objective is to extract crucial global features from complex biological signal data while mitigating the influence of local noise, as depicted in Fig. 1b.

In this framework, the MLP model effectively extracts complex nonlinear features from biological signals using its non-linear activation functions, such as ReLU. It particularly excels in capturing intricate patterns and structures in sEMG and joint angle data during human motion processes^42,43^. Subsequently, Convolutional modules are employed to further diminish the impact of local noise on the representation of global features, thereby enhancing the capability to extract key global features.

The Autoencoder consists of an encoder and a decoder, with the core objective of compressing the input biosignal data into a low-dimensional space (encoding) and then reconstructing the input data through the decoder to faithfully restore the original signals. The encoder comprises two fully connected layers: the first layer compresses the input feature dimension from $input\_dim$ to $input\_dim$/2, and the second layer further compresses it to $input\_dim$/4, with ReLU activation applied in each layer. The decoder symmetrically maps the low-dimensional representation back to the original input dimension. This architecture helps attenuate the influence of local features, extract critical global features from the data, and achieve data compression to some extent. Additionally, the MLP model is easily scalable to deeper or wider network architectures to accommodate complex tasks involving high-dimensional biosignal data. The Autoencoder optimizes denoising performance by adaptively learning features and noise patterns from the input data^44^, without relying on predefined parameters or assumptions, thereby enhancing adaptability to different dataset characteristics, reducing parameter adjustment requirements compared to traditional methods^45^, and improving the efficiency and accuracy of data processing.

Specifically, the autoencoder model structure in this study is described as follows: the input data $E$ is mapped to a low-dimensional latent space representation $D$. $W_1, W_2, W_3, W_4$ represents the weight matrix, and $b_1, b_2, b_3, b_4$ represents the bias vector. input_dim represents the dimension of the input data:

1 $$\begin{aligned} & \text {Encoder}(E) = \text {ReLU}\left( W_2 \cdot \left( \text {ReLU}\left( W_1 \cdot E + b_1\right) \right) + b_2\right) \end{aligned}$$

2 $$\begin{aligned} & W_1 \in \mathbb {R}^{\frac{\text {input}\_\text {dim}}{2} \times \text {input}\_\text {dim}}, \quad W_2 \in \mathbb {R}^{\frac{\text {input}\_\text {dim}}{4} \times \frac{\text {input}\_\text {dim}}{2}} \end{aligned}$$

3 $$\begin{aligned} & b_1 \in \mathbb {R}^{\frac{\text {input}\_\text {dim}}{2}}, \quad b_2 \in \mathbb {R}^{\frac{\text {input}\_\text {dim}}{4}} \end{aligned}$$

4 $$\begin{aligned} & \text {Decoder }(D) = \text {ReLU}\left( W_4 \cdot \left( \text {ReLU}\left( W_3 \cdot D + b_3\right) \right) + b_4\right) \end{aligned}$$

5 $$\begin{aligned} & W_3 \in \mathbb {R}^{\frac{\text {input}\_\text {dim}}{2} \times \frac{\text {input}\_\text {dim}}{4}}, \quad W_4 \in \mathbb {R}^{\text {input}\_\text {dim} \times \frac{\text {input}\_\text {dim}}{2}} \end{aligned}$$

6 $$\begin{aligned} & b_3 \in \mathbb {R}^{\frac{\text {input}\_\text {dim}}{2}}, \quad b_4 \in \mathbb {R}^{\text {input}\_\text {dim}} \end{aligned}$$

### LSTM and attention

Long short-term memory (LSTM)^46^ is considered a classic recurrent neural network structure and is widely used in time series modeling due to its effectiveness in handling long-term dependencies. However, for complex biosignal data such as sEMG and joint angle data, independent LSTM models may have difficulty in fully capturing complex global features and temporal complexity.

The integration of attention mechanism^47^ further enhances the performance of LSTM model in biosignal prediction. Specifically, the attention mechanism enables the model to dynamically learn and assign important weights to data at different stages within the input sequence, thereby more effectively focusing on the most relevant information in prediction tasks. This ability is particularly critical in biosignal processing because biosignal data often exhibits highly dynamic characteristics, including changes in muscle activation patterns and complex changes in joint motion angles^48^. Therefore, the model needs to be able to extract biosignal features that are more useful for predicting joint moments from global features, while ignoring unnecessary local feature information.

By incorporating the attention mechanism, the LSTM model can better capture long-term dependencies and global features when predicting biomechanical parameters such as joint moments. The attention mechanism enables the model to automatically identify and focus on key time steps and features, improving prediction accuracy and stability while reducing interference from irrelevant information. In tasks using sEMG and joint angles, it helps identify critical muscle activation moments or time points related to changes in movement patterns, thereby enhancing the model’s ability to process complex biosignals.

The entire process can be represented by parts (c) and (d) in Fig. 1. The specific implementation process is as follows:

(1) LSTM model:

7 $$\begin{aligned} & I_t = \text {Sigmoid} \left( W_i \cdot t + U_i \cdot H_{t-1} \right) \end{aligned}$$

8 $$\begin{aligned} & F_t = \text {Sigmoid} \left( W_f \cdot t + U_f \cdot H_{t-1} \right) \end{aligned}$$

9 $$\begin{aligned} & O_t = \text {Sigmoid} \left( W_o \cdot t + U_o \cdot H_{t-1} \right) \end{aligned}$$

10 $$\begin{aligned} & \tilde{C}_t = \tanh \left( W_c \cdot t + U_c \cdot H_{t-1} \right) \end{aligned}$$

11 $$\begin{aligned} & C_t = F_t \cdot C_{t-1} + I_t \cdot \tilde{C}_t \end{aligned}$$

12 $$\begin{aligned} & H_t = O_t \cdot \tanh (C_t) \end{aligned}$$

(2) Attention network:

13 $$\begin{aligned} & \text {attention}\_\text {scores} = x \cdot W \end{aligned}$$

14 $$\begin{aligned} & \text {attention}\_\text {weights} = \text {softmax}(\text {attention}\_\text {scores}) \end{aligned}$$

15 $$\begin{aligned} & \text {context}\_\text {vector} = \sum (\text {attention}\_\text {weights} \cdot x) \end{aligned}$$

## Experiment

### Dataset description

The biosignal data used in this study comes from an open source public dataset^49^, which contains fourteen healthy individuals. The EMG signals were acquired using surface electromyography (EMG) sensors (Biometrics Ltd., Newport, UK), with a sampling frequency of 1000 Hz. These sensors were attached to the skin surface to capture the electrical signal of target muscles. Joint angle measurements were performed using Biometrics Ltd. electro-goniometers, also sampled at 1000 Hz, to record the angular variations of the hip, knee, and ankle joints. The individual information is shown in Table 2. The BMI values were calculated using the formula:

16 $$\begin{aligned} \text {BMI} = \frac{\text {Mass (kg)}}{\left( \text {Height (m)}\right) ^2} \end{aligned}$$

and classified according to the following categories: BMI < 18.5: underweight, 18.5 $\le$ BMI < 24.9: normal weight, 25 $\le$ BMI < 29.9: overweight, BMI $\ge$ 30: obese. This dataset was collected by using hardware equipment to obtain the sEMG signals of 11 muscles (tibialis anterior, external oblique, semitendinosus, gluteus medius, rectus femoris, vastus lateralis, vastus medialis, gracilis, biceps femoris , soleus, and gastrocnemius medialis) of the subjects in three gaits (treadmill, level-ground, and ramp). The hip adduction joint angle and hip flexion joint angle as well as the hip flexion/extension (Hip FE) and hip abduction/adduction (Hip AA) moment are calculated through Opensim. In addition, each subject performed 7 trials on the treadmill, with a total of 28 speeds. Each trial consisted of accelerating to a constant speed and then decelerating. For example, the speeds of the first treadmill trial were 0.5 m/s, 1.2 m/s, 1.55 m/s, and 0.85 m/s, respectively. All speeds were increased by 0.05 m/s in each subsequent trial (such as the second trial was performed at 0.55 m/s, 1.25 m/s, 1.6 m/s, and 0.9 m/s), and each speed was maintained for 30 s between each acceleration. During level-ground gait, the mean speeds for all subjects were 0.88 ± 0.19 m/s (slow self-selected speed), 1.17 ± 0.21 m/s (normal speed), and 1.45 ± 0.27 m/s (fast speed). The ramp test for each subject was performed on a 5-m-long ramp with six inclination angles of $5.2^{\circ }$, $7.8^{\circ }$, $9.2^{\circ }$, $11^{\circ }$, $12.4^{\circ }$, and $18^{\circ }$. Similar to stair climbing, subjects completed a set of 5 trials for each starting leg on each ramp, for a total of 60 ramp trials. Due to the small amount of data for level-ground and ramp gaits, as well as the problem of missing data, the experiments in this study were mainly conducted based on treadmill gait data.

**Table 2:** Physical characteristics and BMI of each subject.

Subject ID	Age	Gender	Height (cm)	Mass (kg)	BMI	Health status
1	20	Male	165	55.3	20.3	Normal weight
2	21	Male	174	72.6	24.0	Normal weight
3	21	Female	163	63.5	23.9	Normal weight
4	22	Male	175	83.9	27.4	Overweight
5	21	Male	175	77.1	25.1	Overweight
6	24	Male	174	86.2	28.5	Overweight
7	22	Female	152	58.4	25.3	Overweight
8	20	Female	165	55.8	20.5	Normal weight
9	19	Female	180	60.1	18.5	Normal weight
10	21	Female	171	68.0	23.3	Normal weight
11	21	Female	173	72.6	24.2	Normal weight
12	20	Female	163	52.2	19.6	Normal weight
13	22	Male	170	68.0	23.5	Normal weight
14	31	Male	177	77.0	24.6	Normal weight

The BMI values indicate the health status: BMI < 18.5: Underweight, 18.5 $\le$ BMI < 24.9: Normal weight, 25 $\le$ BMI < 29.9: Overweight, BMI $\ge$ 30: Obese.

### Data preprocessing

During the data processing, the sampling rate of the sEMG signals of each subject in the dataset was 1000 Hz, while the sampling rates of the hip adduction joint angle and hip flexion joint angle as well as the Hip FE moment and Hip AA moment were 200 Hz. In order to arrange the experiments in a reasonable time sequence, the sEMG signals were downsampled in this study in each gait experiment with a time step of 5 ms to reduce its sampling frequency to 200 Hz. Subsequently, the downsampled data was further downsampled to 20 Hz for treadmill gait data to facilitate model simulation of real-time detection applications. Specifically, based on the hip joint moment drive principle^50^, muscle signals (Gluteus medius, Semitendinosus, Biceps femoris, Rectus femoris) and hip adduction joint angle and hip flexion joint angle were selected as input data for predicting Hip FE and Hip AA. Considering the individual differences between subjects, this study trained a model for each individual separately. In order to emphasize the generalization ability of the model, the first experiment of each individual’s treadmill gait (such as (0.5 m/s, 1.3 m/s, 1.7 m/s, 0.9 m/s)) is used as the training set, and the remaining 6 experiments are used as the test set. Due to the lack of data in level-ground and ramp gait, this study will take 80% of the data as the training set and 20% as the test set.

**Fig. 2:**
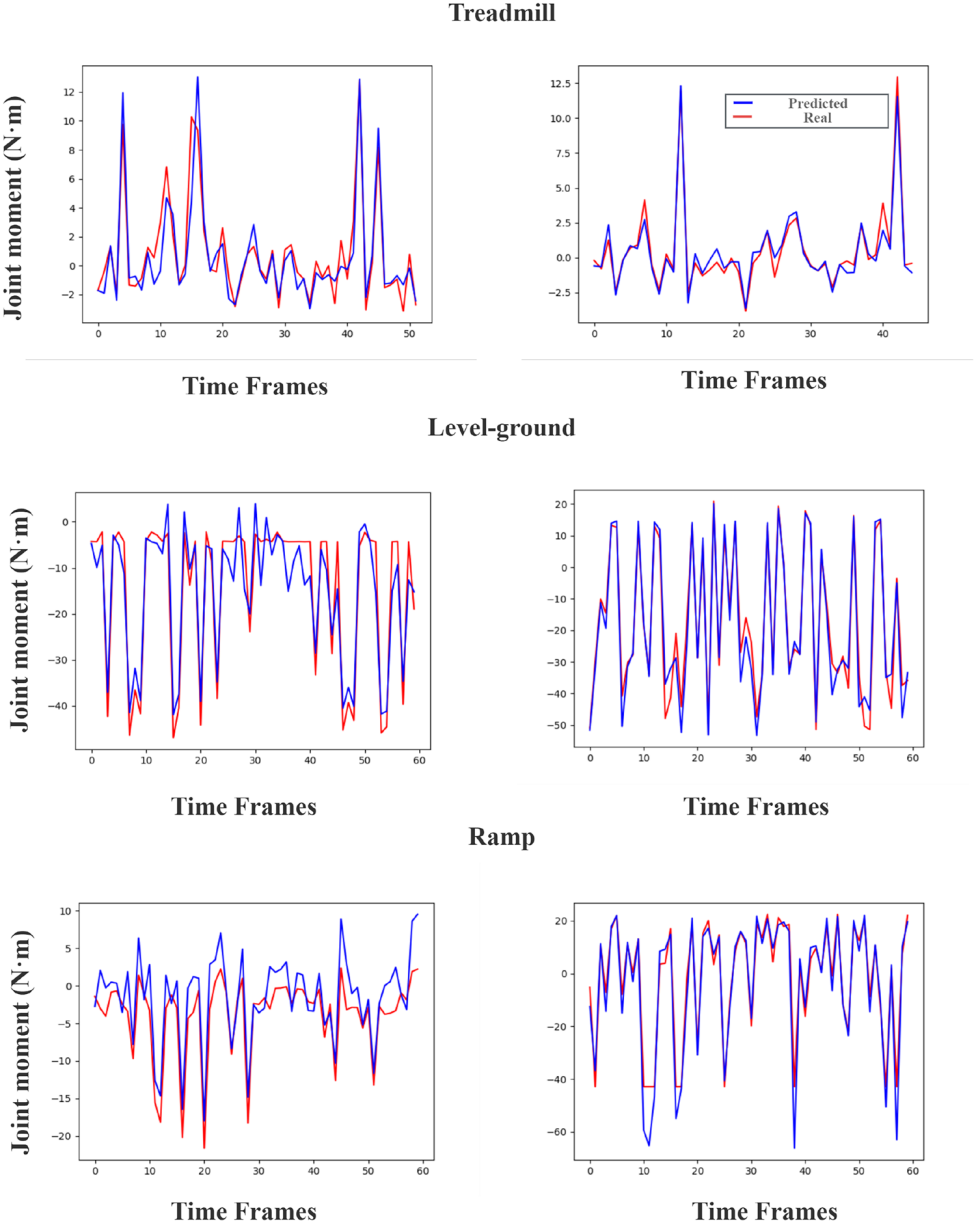
Typical prediction results of the LSTAM model in fourteen subjects, where the blue lines represent the joint moments calculated by the inverse dynamics method and the red lines represent the predicted values from our model.

### Performance metrics for evaluation

The prediction of joint moment in this study belongs to the regression task, so in order to evaluate the prediction accuracy of the model in the experiment, three evaluation indicators were selected in this study: root mean square error (RMSE), coefficient of determination ($\hbox {R}^{2}$) and variance accounted for (VAF). The formulas are shown as follows:

17 $$\begin{aligned} & R M S E=\sqrt{\sum _{i}^{N} \frac{\left( p_i-\hat{p}_i\right) ^{2}}{N}} \end{aligned}$$

18 $$\begin{aligned} & R^2 =1 - \frac{{\textstyle \sum _{i=1}^{N}{\left( p_i-\hat{p}_i\right) ^{2}}}}{{\textstyle \sum _{i=1}^{N}{\left( p_i-\bar{p}\right) ^{2}}}} \end{aligned}$$

19 $$\begin{aligned} & VAF= \left[ 1- \frac{var\left( \hat{p}_i-p_i \right) }{var\left( p_i \right) }\right] \end{aligned}$$

where $N$ represents the number of data points, $p_i$ is the actual value, $\hat{p}_i$ is the predicted value, and $\bar{p}$ is the average of the actual values.

In these indicators, RMSE measures the prediction error of the model by working out the root mean square difference between the model prediction value and the actual observation value. Therefore, a lower RMSE value is considered more favorable, indicating that the model has a smaller prediction error. $\hbox {R}^{2}$ is a measure used to evaluate the goodness of fit of the model, which represents the percentage of the dependent variable variance that the model can explain. A higher $\hbox {R}^{2}$ value indicates that the model fits the actual data better. VAF is a measure used to quantify the proportion of the total variance in the data that the model can explain. A higher VAF value is also considered more desirable, indicating that the model has an enhanced ability to describe the data.

**Fig. 3:**
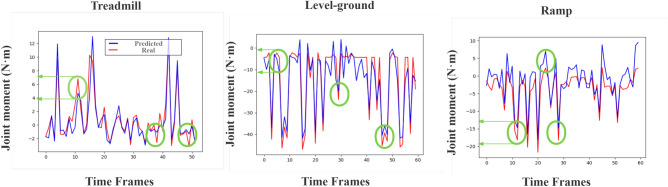
The discrepancy between the predicted and actual joint moments, with the green circles highlighting regions of larger deviations. It can be observed that the prediction error remains within 5 N$\cdot$m during treadmill gait, while for level-ground and ramp gait, the error is maintained within 10 N$\cdot$m, demonstrating the model’s stable predictive performance across different gait patterns.

### Experimental setting

In this study, the Python programming language was used to build the model based on the PyTorch framework, and the Adam optimizer was used for training on the NVIDIA A40-40G GPU. The input window size was set to 300 ms, which corresponded to 60 samples after downsampling. The MLP module was initialized with 60 input neurons, consistent with the input dimension, and the convolutional module used the same window size for feature extraction. A single-layer LSTM network was employed to capture temporal dependencies, with a batch size of 60. The learning rate of the Adam optimizer was set to 0.001. The loss function was the MSE, and a dropout rate of 0.2 was applied to prevent overfitting. The number of epochs was set to 30, and the input remained consistent during training.

## Results

### Model feasibility

To further evaluate the performance of the model, the first experimental data of each subject on the treadmill was used for training, and the joint moment of the remaining six experimental data of the subject was predicted. The data in Table 3 show that the average prediction results of the model for each individual under different speed conditions had high accuracy.

**Table 3:** The performance of the LSTAM model for fourteen subjects using VAF, RMSE, and $\hbox {R}^{2}$ metrics to evaluate 6 test sets in treadmill gait.

Subject ID	Hip FE			Hip AA		
	VAF	RMSE	$\hbox {R}^{2}$	VAF	RMSE	$\hbox {R}^{2}$
Subject 1	0.904 ± 0.022	11.328 ± 2.440	0.896 ± 0.023	0.935 ± 0.007	5.087 ± 0.318	0.933 ± 0.007
Subject 2	0.881 ± 0.009	9.754 ± 1.084	0.877 ± 0.011	0.910 ± 0.024	6.082 ± 0.684	0.906 ± 0.026
Subject 3	0.906 ± 0.014	7.369 ± 1.121	0.903 ± 0.015	0.940 ± 0.007	4.951 ± 0.366	0.938 ± 0.007
Subject 4	0.891 ± 0.019	11.836 ± 2.012	0.887 ± 0.018	0.900 ± 0.018	6.868 ± 0.620	0.896 ± 0.016
Subject 5	0.891 ± 0.024	9.606 ± 2.085	0.884 ± 0.028	0.920 ± 0.025	5.682 ± 0.943	0.918 ± 0.026
Subject 6	0.881 ± 0.015	9.742 ± 1.571	0.876 ± 0.016	0.918 ± 0.008	8.248 ± 0.243	0.895 ± 0.011
Subject 7	0.936 ± 0.011	5.162 ± 0.835	0.935 ± 0.011	0.943 ± 0.014	4.156 ± 0.550	0.942 ± 0.014
Subject 8	0.879 ± 0.017	7.394 ± 1.205	0.876 ± 0.017	0.909 ± 0.013	4.117 ± 0.362	0.909 ± 0.013
Subject 9	0.933 ± 0.009	6.344 ± 0.663	0.931 ± 0.010	0.917 ± 0.015	4.103 ± 0.551	0.908 ± 0.022
Subject 10	0.941 ± 0.006	5.489 ± 0.592	0.960 ± 0.006	0.968 ± 0.005	3.232 ± 0.295	0.968 ± 0.006
Subject 11	0.915 ± 0.003	5.634 ± 0.443	0.953 ± 0.005	0.969 ± 0.004	4.258 ± 0.317	0.968 ± 0.004
Subject 12	0.942 ± 0.008	4.897 ± 0.504	0.941 ± 0.008	0.965 ± 0.005	2.759 ± 0.273	0.964 ± 0.006
Subject 13	0.891 ± 0.049	10.073 ± 1.728	0.887 ± 0.055	0.898 ± 0.013	8.943 ± 1.153	0.868 ± 0.428
Subject 14	0.911 ± 0.014	7.858 ± 1.939	0.907 ± 0.038	0.887 ± 0.054	9.342 ± 1.500	0.896 ± 0.331

The mean and standard deviation of these metrics are used to highlight the accuracy of the model in predicting Hip FE and Hip AA.

The LSTAM model uses sEMG signals, hip adduction angle, and hip flexion angle as input variables to predict Hip FE and Hip AA for each subject. The model assigns importance weights through dynamic learning, focuses on the extraction of global features, and weakens the influence of unnecessary data such as local features (including noise). The mean and standard deviation of the three indicators of VAF, RMSE, and $\hbox {R}^{2}$ were used as evaluation indicators. The results show that the model has high consistency and accuracy in interpreting nonlinear biosignal data in different test instances, as illustrated in Fig. 2. This indicates that the model is highly adaptable in dealing with the trend of joint torque changes, and has good generalization ability and robustness for dynamic and complex biomechanical environments.

In addition, this study also plans to conduct ablation experiments on the LSTAM model, specifically using treadmill gait data to fully verify the effectiveness of the LSTAM model in extracting global features of nonlinear signals. The ablation experiment will gradually remove the influence of the MLP module, attention mechanism, and convolution module in the model. By gradually removing these key components, we aim to evaluate the necessity and effectiveness of each module in processing nonlinear biological signals to clarify the contribution of each part to the overall performance of the model. In particular, the MLP module and the convolution module play an important role in eliminating local features and blurring detail features in the signal, which is crucial for accurately extracting important features in biological signals. The attention mechanism helps the model to focus on relevant signal features more effectively when dealing with long-term dependencies, thereby improving prediction accuracy.

The following is an introduction to the ablation model:

(1) *Model I*: The original LSTM model.

(2) *Model II*: The model eliminates the MLP module and FFT transformation and uses the attention mechanism.

(3) *Model III*: The model that eliminates the attention module but performs FFT processing and uses the MLP module.

(4) *Model IV*: The model with MLP module and FFT transform combined with attention mechanism module.

(5) *Model V*: The complete LSTAM model proposed in this study includes FFT processing as well as MLP module, convolution module, and attention mechanism.

Table 4 shows the results of the ablation experiment. From the data of predicting the two indicators of Hip FE and Hip AA, it can be seen that the LSTAM (Model V) proposed in this study performs the best on multiple evaluation criteria, highlighting the significant advantage of the LSTAM model in processing the global features of complex biological signal data to make accurate predictions. Specifically, the LSTAM model enables the model to focus on most of the global features that are beneficial to the prediction results, reduce the misleading of random fluctuations or noise in local features, capture complex time dependencies and complex patterns, and achieve the optimal values in VAF and RMSE when predicting Hip FE and Hip AA, showing its comprehensive advantages in prediction tasks at different speeds.

**Table 4:** The performance of the three ablation models and the LSTAM model on fourteen subjects, using VAF, RMSE, and $\hbox {R}^{2}$ metrics to evaluate 6 test sets in treadmill gait.

Model	Hip FE			Hip AA		
	VAF	RMSE	$\hbox {R}^{2}$	VAF	RMSE	$\hbox {R}^{2}$
Model I	0.846 ± 0.090	9.47 ± 3.35	0.837 ± 0.093	0.873 ± 0.058	8.78 ± 6.28	0.781 ± 0.309
Model II	0.854 ± 0.067	9.57 ± 2.96	0.845 ± 0.069	0.887 ± 0.051	8.41 ± 5.80	0.774 ± 0.279
Model III	0.848 ± 0.041	8.90 ± 1.59	0.841 ± 0.040	0.890 ± 0.050	8.27 ± 5.64	0.820 ± 0.264
Model IV	0.890 ± 0.052	8.78 ± 2.78	0.883 ± 0.053	0.918 ± 0.045	7.46 ± 5.54	0.797 ± 0.278
**Model V**	**0.907** ± **0.022**	**8.04** ± **2.27**	**0.908** ± **0.029**	**0.927** ± **0.026**	**5.56** ± **2.01**	**0.922** ± **0.030**

The mean and standard deviation of these metrics are used to highlight the accuracy of the model in predicting Hip FE and Hip AA. The results in bold represent the best.

### Comparative experiments

In order to highlight the superiority of the model proposed in this study, we compared it with the current mainstream biosignal prediction models. As comparison models, we selected the long short-term memory network (LSTM)^46^ and the temporal convolutional network (TCN)^51^, Conv2d^13^, FFN^52^, TimeMixer^53^,xPatch^54^, TransEMG^28^, and trained and tested them using the same experimental settings as LSTAM. Table 5 shows the comparison results in treadmill gait prediction, and Table 6 shows the comparison results in level-ground (LG) and ramp gait. It can be clearly observed from these two tables that the LSTAM model is significantly better than the LSTM and TCN models in predicting Hip FE and Hip AA. Specifically, the LSTAM model performs well in the three key indicators of VAF, $\hbox {R}^{2}$ and RMSE, and its prediction accuracy is better than the comparison models. This shows that the LSTAM model can better extract the global features of biosignals and more accurately capture the changes and fluctuations of biosignals.

## Discussion

Considering that when the human body is running with the equipment attached to the human skin, it will cause the sEMG signal to fluctuate, so that the features extracted by the model contain unnecessary parts, and the human body will affect muscle activation to varying degrees when running, resulting in a sharp change in joint moment. This study has achieved remarkable results in predicting joint moments using nonlinear signals at different speeds in treadmill gait exploration, and improved the utilization rate of global features. Under the condition of sufficient treadmill gait data, the data is not preprocessed for noise removal. Each subject only uses one treadmill gait experiment data (using sEMG signals and joint angle data) to train the LSTAM model. In the other 6 experiments (24 speeds) of each subject, good Hip FE and Hip AA prediction effects were obtained. As shown in Table 3, the three indicators (VAF, RMSE, $\hbox {R}^{2}$) all performed well, demonstrating the model’s ability to handle nonlinear signal feature extraction.

LSTM excels at capturing long-term dependencies in time series prediction and performs well in the tasks of this study, as shown in Tables 4, 5, and 6. The LSTAM model builds on LSTM for biological signals, emphasizing global feature utilization while reducing the influence of noisy or less relevant local features. Although it achieves good accuracy with few iterations, it has not fully leveraged inverse dynamics or human structural characteristics. From the ablation experiment results shown in Table 4, it can be seen that the improvement of the model in reducing the influence of local features has a significant effect on the prediction performance. Specifically, in Model II, after the introduction of the attention mechanism, the three evaluation indicators (VAF, RMSE and $\hbox {R}^{2}$) of the model on Hip FE and Hip AA are better than the original LSTM model. This shows that by enhancing the model’s ability to extract key features in biological signals, the model’s utilization efficiency of global features can be effectively improved. In Model III, we use MLP combined with FFT to weaken the influence of local features without using the attention mechanism. The results show that compared with the LSTM model, Model III has improved in the three evaluation indicators, but still has shortcomings in extracting joint features. Model IV combines the attention mechanism, MLP and FFT processing methods, so that the model can more effectively focus on the key information in the global features while weakening the influence of local features. The experimental results show that Model IV is better than Model I, Model II and Model III in all three evaluation indicators, indicating that this comprehensive method has significant advantages in extracting and utilizing global features. In order to further improve the model’s efficiency in utilizing global features, a convolution module is also introduced in Model IV, which further enhances the performance of the model by blurring local features.

**Table 5:** The performance of the comparison model on fourteen subjects, using VAF, RMSE, and $\hbox {R}^{2}$ metrics to evaluate 6 test sets in treadmill gait.

Model	Hip FE			Hip AA		
	VAF	RMSE	$\hbox {R}^{2}$	VAF	RMSE	$\hbox {R}^{2}$
LSTM	0.846 ± 0.090	9.47 ± 3.35	0.837 ± 0.093	0.873 ± 0.058	8.78 ± 6.28	0.781 ± 0.309
TCN	0.771 ± 0.084	10.74 ± 3.15	0.756 ± 0.089	0.852 ± 0.067	8.82 ± 4.77	0.768 ± 0.262
TimeMixer	0.758 ± 0.081	11.02 ± 3.08	0.742 ± 0.085	0.841 ± 0.072	9.12 ± 4.96	0.752 ± 0.271
xPatch	0.889 ± 0.034	9.18 ± 2.64	0.886 ± 0.038	0.891 ± 0.024	6.39 ± 2.41	0.895 ± 0.046
Conv2D	0.886 ± 0.037	9.27 ± 2.78	0.882 ± 0.041	0.884 ± 0.021	6.51 ± 2.56	0.888 ± 0.049
FFN	0.805 ± 0.079	10.09 ± 3.18	0.802 ± 0.077	0.793 ± 0.081	9.85 ± 3.62	0.766 ± 0.142
TranSEMG	0.864 ± 0.072	9.52 ± 2.98	0.854 ± 0.075	0.909 ± 0.040	7.78± 5.22	0.896 ± 0.240
**LSTAM**	**0.907** ± **0.022**	**8.04** ± **2.27**	**0.908** ± **0.029**	**0.927** ± **0.026**	**5.56** ± **2.01**	**0.922** ± **0.030**

The mean and standard deviation of these metrics are used to highlight the accuracy of the model in predicting Hip FE and Hip AA. The results in bold represent the best.

**Table 6:** The mean and standard deviation of the compared models for LG and ramp gait using VAF, RMSE, and $\hbox {R}^{2}$ metrics to highlight the accuracy of the model in predicting Hip FE and Hip AA.

Scene	Model	Hip FE			Hip AA		
		VAF	RMSE	$\hbox {R}^{2}$	VAF	RMSE	$\hbox {R}^{2}$
LG	LSTM	0.932 ± 0.018	7.42 ± 2.35	0.919 ± 0.041	0.871 ± 0.032	5.48 ± 1.32	0.858 ± 0.039
	TCN	0.918 ± 0.047	9.61 ± 3.92	0.861 ± 0.098	0.541 ± 0.031	6.64 ± 5.87	0.512 ± 0.214
	TimeMixer	0.903 ± 0.052	10.21 ± 4.11	0.842 ± 0.104	0.518 ± 0.028	6.98 ± 6.13	0.486 ± 0.231
	Conv2D	0.768 ± 0.028	6.85 ± 1.94	0.762 ± 0.031	0.801 ± 0.018	7.46 ± 2.21	0.796 ± 0.027
	xPatch	0.774 ± 0.025	6.73 ± 1.88	0.769 ± 0.029	0.808 ± 0.016	7.31 ± 2.04	0.804 ± 0.025
	FFN	0.756 ± 0.046	8.52 ± 1.97	0.752 ± 0.058	0.748 ± 0.039	8.71 ± 2.41	0.744 ± 0.064
	TranSEMG	0.947 ± 0.019	6.02 ± 1.11	0.938 ± 0.014	0.901 ± 0.014	5.88 ± 1.01	0.903 ± 0.017
	**LSTAM**	**0.958** ± **0.007**	**5.61** ± **1.12**	**0.942** ± **0.015**	**0.916** ± **0.036**	**4.32** ± **1.71**	**0.908** ± **0.042**
Ramp	LSTM	0.937 ± 0.006	6.28 ± 0.58	0.934 ± 0.006	0.678 ± 0.038	3.21 ± 0.19	0.643 ± 0.019
	TCN	0.948 ± 0.004	9.82 ± 2.31	0.836 ± 0.059	0.572 ± 0.027	4.33 ± 0.41	0.404 ± 0.081
	TimeMixer	0.941 ± 0.006	10.34 ± 2.58	0.821 ± 0.064	0.549 ± 0.024	4.57 ± 0.46	0.382 ± 0.087
	Conv2D	0.872 ± 0.018	10.06 ± 1.14	0.861 ± 0.026	0.646 ± 0.028	4.51 ± 1.07	0.641 ± 0.029
	xPatch	0.879 ± 0.016	9.92 ± 1.09	0.868 ± 0.024	0.652 ± 0.026	4.38 ± 0.98	0.647 ± 0.027
	FFN	0.826 ± 0.036	11.24 ± 1.87	0.811 ± 0.028	0.481 ± 0.025	7.64 ± 1.84	0.482 ± 0.031
	TranSEMG	0.953 ± 0.009	5.14 ± 0.54	0.954 ± 0.011	0.701 ± 0.021	4.08 ± 2.03	0.637 ± 0.024
	**LSTAM**	**0.963** ± **0.013**	**5.02** ± **0.71**	**0.960** ± **0.010**	**0.719** ± **0.014**	**3.36** ± **0.35**	**0.661** ± **0.067**

The results in bold represent the best.

When dealing with LG and ramp gait, due to limited data and missing values (Table 6), the model’s performance in predicting Hip AA is lower than that in treadmill gait. For instance, TCN achieves VAFs of only 0.541 and 0.572 for LG and ramp Hip AA, respectively, while LSTAM reaches 0.916 and 0.719. TimeMixer and FFN also show relatively poor results, highlighting the impact of small sample sizes and missing data on model fitting. Although LSTAM still outperforms other models across metrics, it cannot fully address missing data. Future work could leverage biological signal augmentation or diffusion models to enrich the dataset, thereby improving global feature utilization and better capturing human kinematic patterns. Moreover, the experimental settings and model configurations used in this study are not universally applicable to all individuals or more complex gait patterns. To extend the model to other applications, it may be necessary to adjust model parameters, modify the number of MLP layers, or optimize the attention mechanism. Such adjustments help ensure that the model maintains high performance across diverse conditions and populations while accounting for variations in signal quality and noise.

The LSTAM model proposed in this study demonstrates excellent performance in joint moment prediction, as illustrated in Fig. 3, providing important theoretical and methodological support for human rehabilitation assessment. By incorporating FFT frequency-domain decomposition and attention mechanisms, the model can more accurately capture the global features of joint moments while reducing the interference of local noise, thereby improving the accuracy of rehabilitation assessments. Its outcomes can be directly applied to the control systems of intelligent rehabilitation devices, such as exoskeleton robots and smart prosthetics, enabling these devices to more naturally simulate human motion and enhancing patient rehabilitation outcomes and quality of life. Although LSTAM includes multiple computational components, the use of efficient 1D convolution, MLP, and FFT operations keeps the overall inference cost manageable. With optimized implementation, the model can run in real time on modern embedded processors or wearable platforms, making its application in practical wearable rehabilitation devices feasible.

Despite the strong performance of the LSTAM model in joint moment prediction, several limitations remain. The current experiments mainly involved healthy adult participants with a limited sample size, so the model’s generalizability to different age groups, pathological gaits, or clinical populations still needs to be validated. Data were primarily collected under treadmill and level/ramp walking conditions, and the model’s performance in more complex real-world environments requires further evaluation. Although attention mechanisms and frequency-domain analysis are incorporated, the weighting of specific frequency bands or temporal phases has not been fully interpreted, leaving room to improve clinical interpretability. In addition, real-time deployment on wearable devices requires further optimization, including model compression and hardware acceleration. Finally, the current study only considers sEMG, joint angles, and joint moments, and integration with other physiological signals remains a promising direction for future research. Future work can explore model compression and hardware acceleration to further improve real-time performance while maintaining prediction accuracy. In addition to improving real-time performance, the increasing availability of diverse healthcare and biosignal data motivates the exploration of multimodal data fusion for more comprehensive analysis. With the increasing availability of diverse healthcare and biosignal data, such as EMG, joint angles, joint moments, medical images, and electronic health records, there is a growing need to effectively integrate and fuse these multimodal data for comprehensive analysis and decision-making. However, multimodal data fusion in healthcare remains challenging due to the heterogeneity of data types and the complexity of selecting appropriate fusion strategies. Teoh et al.^55^ provided a comprehensive review of recent techniques for multimodal healthcare data fusion, summarizing various integration methods, early/intermediate/late fusion strategies, as well as their associated challenges and potential applications. In this context, the LSTAM model can effectively handle multimodal biological signals (such as EMG, joint angles, and joint moments), offering new approaches for multimodal data fusion analysis and laying the foundation for multidimensional data analysis. By combining global and local feature learning, LSTAM can better adapt to individual differences among patients, providing technical support for the development of personalized rehabilitation plans. Furthermore, the model and methods proposed in this study offer tools for standardizing and automating rehabilitation assessments, helping reduce human errors and improving the consistency and reliability of evaluations. Finally, the innovative integration of frequency-domain analysis and attention mechanisms provides new directions for future research, such as incorporating additional biological signals like EEG or heart rate into joint moment prediction to enhance the comprehensiveness of rehabilitation assessments.

## Conclusion

This study proposes the LSTAM model as a novel approach for joint moment prediction based on biosignals. By integrating FFT frequency-domain decomposition with MLP and convolutional modules, the model attenuates the interference of local features in nonlinear biosignals while enhancing the extraction and updating of key global features, thereby significantly improving the efficiency of feature utilization. Experimental results demonstrate that the LSTAM model outperforms several state-of-the-art methods, showing excellent predictive performance. Given its advantages in processing nonlinear biosignals, the LSTAM model holds great potential for human rehabilitation assessment. Future research will focus on further improving the model’s generalization capability in more complex and diverse scenarios, broadening its applicability and ensuring robustness.

## Data Availability

The Camargo biomechanics dataset used in this study is publicly available at http://www.epic.gatech.edu/opensource biomechanicscamargo-et-al/. The data are organized as tables following a nested directory structure of subject/date/mode/sensor.
